# From food deserts to nutritional equity: exposing socioeconomic drivers of hypertension

**DOI:** 10.1017/jns.2025.10067

**Published:** 2026-01-13

**Authors:** Zihao Yi, Masoud Khani, Mohammad Assadi Shalmani, Amirsajjad Taleban, Jennifer T. Fink, Robert F. Frediani, Jake Luo

**Affiliations:** 1 Joseph J. Zilber College of Public Health, University of Wisconsin Milwaukee, Milwaukee, WI, USA; 2 School of Biomedical Science and Health Care Administration, University of Wisconsin Milwaukee, Milwaukee, WI, USA

**Keywords:** Cardiovascular health disparities, Food environment, Hispanic paradox, Social determinants of health, Health equity, Food deserts, Hypertension prevalence

## Abstract

This study investigates the associations between social determinants of health (SDOH) and hypertension prevalence across Wisconsin communities, with particular attention to food environments, economic factors, and transportation patterns. Using data from the 2019–2020 Wisconsin State Inpatient Database (387,047 patients) and the 2020 AHRQ SDOH database, we employed spatial analysis and logistic regression models to examine relationships between hypertension prevalence and neighbourhood characteristics across 597 ZIP codes. Lower-income areas exhibited significantly higher hypertension prevalence (EE = 1.233, 95% CI: 1.128–1.347 for incomes under $14,999), neighbourhoods with greater food resource density showed protective associations (EE = 0.549, 95% CI: 0.474–0.636 for supermarket access). Active transportation patterns were associated with lower hypertension rates (EE = 0.879, 95% CI: 0.829–0.933 for walking). We observed a ‘Hispanic paradox’ in Milwaukee County, where Hispanic populations demonstrated lower hypertension prevalence despite socioeconomic disadvantages, whereas African American populations with similar disadvantages exhibited higher prevalence. Our proposed ‘Food Environment Synergy Model’ helps frame these findings by conceptualising food environments through three interacting dimensions: physical access, economic accessibility, and cultural dietary patterns. This integrated approach highlights how these dimensions collectively relate to unique risk and resilience profiles within communities, challenging conventional binary classifications of ‘food deserts’ versus ‘food secure’ areas. These findings indicate that addressing food access disparities, promoting walkable neighbourhoods, and preserving beneficial cultural dietary traditions may be related to lower hypertension prevalence and advance health equity in diverse communities. However, the analysis is cross-sectional, causality cannot be inferred; further longitudinal studies are needed to establish causal relationships.

## Introduction

Hypertension remains one of the most pressing global health challenges and is strongly associated with the burden of cardiovascular disease (CVD), stroke, kidney failure, and premature mortality.^(1,2)^ As a leading modifiable risk factor, it affects nearly one-third of adults worldwide and disproportionately impacts populations in low-income and resource-constrained settings.^(3)^ Although clinical advancements have significantly improved the treatment and prevention of hypertension, persistent gaps remain in understanding how environmental and socioeconomic factors drive its prevalence underscoring the urgent need for deeper investigation. Specifically, the ways in which patients’ living environments influence the development and progression of hypertension remain inadequately explored, leaving critical questions unanswered about the intersection of population health gaps and chronic disease outcomes.^(3,4)^

Recent studies have worked on characterising neighbourhood food environments through binary classifications of ‘food deserts’ or ‘food secure’ areas.^(5,6)^ These investigations have documented associations between limited food access and adverse cardiovascular outcomes, with Kelli et al.^(5)^ reporting a 1.58-fold increased hazard ratio for cardiovascular events among patients living in food deserts. However, these binary classifications often fail to capture the complex interplay between food resource availability, economic accessibility, and cultural dietary patterns that collectively are associated with cardiovascular risk profiles. Further research by Testa et al.^(6)^ examining only supermarket density without considering broader food environment characteristics has yielded inconsistent results, highlighting the need for more nuanced analytical frameworks.

Social determinants of health (SDOH), which refer to non-medical factors that are associated with health and well-being, have emerged as pivotal contributors to disparities in disease prevalence and outcomes.^(7,8)^ These determinants encompass socioeconomic status, education, access to healthcare, neighbourhood environments, and social networks, all of which relate to health behaviours, resource access, and exposure to risks.^(9)^ Increasingly recognised as fundamental determinants associated with of health inequities, SDOH plays a particularly significant role in chronic diseases such as hypertension.^(10,11)^ However, despite growing attention in public health research, the direct relationship between these determinants and hypertension prevalence, particularly when examined through a multidimensional framework, remains insufficient.

Emerging evidence increasingly links social determinants of health (SDOH), including income, food access, and transportation, to hypertension outcomes; however, most existing studies examine these factors in isolation, overlooking their interconnected and compounding effects.^(4)^ Living in food deserts, reliance on highly processed foods, and economic barriers to accessing healthcare have been identified as factors associated with higher hypertension prevalence in disadvantaged populations.^(5,12)^ However, the complex interplay of these determinants, including their geographic, cultural, and economic dimensions, remains poorly understood. Further, it is unclear how these factors collectively are associated with disparities in hypertension prevalence among urban, suburban, and rural communities.^(13)^

Few studies have comprehensively examined the relationship between specific neighbourhood characteristics, such as food resource availability, income inequality, and transportation options, and hypertension prevalence.^(10)^ This study addresses this gap by investigating the associations between SDOH and hypertension across diverse settings in Wisconsin, utilising spatial analysis, heatmaps, and patient-level data. By highlighting how SDOH factors interact to are associated with hypertension prevalence, our findings provide actionable insights for targeted interventions. This research underscores the importance of addressing socioeconomic and environmental disparities in public health to address disparities related to the burden of hypertension and promote equitable health outcomes.^(14–16)^

## Method

### Study design and data sources

We conducted a cross-sectional study to examine the associations between hypertension prevalence and social determinants of health (SDOH) across Wisconsin communities, with a focused analysis on Milwaukee County. Data were sourced from the State Inpatient Databases (SID) for 2019–2020 and the Agency for Healthcare Research and Quality (AHRQ) SDOH database for 2020. The SID dataset provided patient-level information, including hypertension diagnoses (ICD-10 code I10), demographic characteristics, and hospitalisation records, while the AHRQ dataset supplied ZIP code-level variables such as income distribution, transportation patterns, racial composition, and food resource availability. These datasets were integrated using ZIP codes as the common geographic identifier to examine the relationships of SDOH factors on hypertension prevalence.

### Variables and measures

The primary outcome variable was hypertension prevalence, defined as the proportion of patients diagnosed with hypertension in each ZIP code and normalised by the total weighted population. Independent variables included income levels categorised into quartiles derived from the SID database and specific income brackets from the SDOH data (‘Income under $10,000’ to ‘Income over $100,000’). Other variables included food resource density (convenience stores, supermarkets, fast-food outlets, and full-service restaurants per 1000 residents), transportation patterns (walking, driving, or public transit to work), racial and ethnic composition (White, Black, Hispanic, Asian, Native American, and Other Race), and food stamp usage. The dataset comprised 597 ZIP codes and 387,047 patients after filtering for Wisconsin residents aged 20 years or older. Milwaukee County, with 76,403 patients, was analysed separately to examine localised urban disparities.

### Data integration and analysis

Data preprocessing involved merging the SID and AHRQ datasets using ZIP codes and filtering for patients aged 20 and older with valid ZIP codes. Cases with missing age or ZIP code data and ZIP codes outside Wisconsin were excluded. Missing values in the SDOH data were imputed using the k-nearest neighbours (KNN) algorithm, resulting in a final dataset of 387,047 cases. Descriptive analyses summarise hypertension prevalence and SDOH characteristics across ZIP codes. Pearson correlation coefficients evaluated associations between SDOH variables and hypertension prevalence, while logistic regression models were used to examine the associations of SDOH factors, with results reported as effect estimates (per 1% increase) together with 95% confidence intervals. Heatmaps visualised geographic distributions of hypertension prevalence and SDOH variables. All analyses were conducted in Python 3, utilising libraries including pandas, numpy, matplotlib, seaborn, and statsmodels for statistical computations and visualisations.

## Result

### Social-demographic and patient characteristics

The study sample included 387,047 patients diagnosed with hypertension in Wisconsin from the 2019–2020 State Inpatient Database. As detailed in Table 1, significant differences were observed in patient distribution by age, gender, and race.

**Table 1.** Demographic characteristics, hypertension prevalence rates, and odds ratios among patients (*n* = 387,047)

Variables	Patient number	Prevalence rate	Proportion	Odds ratio	*p*-Value
**Age**					
20–29 (Ref)	3899	0.265%	1.01%	1.00	1.00
30–44	26,938	1.784%	6.96%	6.83	<0.01
45–64	13,9921	6.560%	36.15%	26.40	<0.01
65+	21,6289*	16.17%*	55.88%*	72.6*	<0.01
Total	387,047	6.000%	100%	N/A	N/A
**Gender**					
Male (Ref)	185,228	4.533%	47.86%	1.00	1.00
Female	**203,427**	4.963%*	52.56%*	1.10*	<0.01
**Race**					
White (Ref)	335,280*	4.861%	86.63%*	1.00	1.00
Black	337,22	7.143%*	8.71%	1.51*	<0.01
Hispanic	122,48	1.919%	3.16%	0.38	<0.01
Asian	3379	1.350%	0.87%	0.27	<0.01
Native American	3542	5.337%	0.92%	1.10	<0.01
Other	491	0.230%	0.13%	0.05	<0.01

Mean values were significantly different from those of the reference group: *p < 0.01.

OR, odds ratio; Ref, reference group.

Superscript symbol (*) denotes significant differences compared with reference groups within each category (*p* < 0.01).

Overall, the majority of patients were female (*n* = 203,427; 52.56%), White (*n* = 335,280; 86.63%), and aged 65 years or older (*n* = 216,289; 55.88%). Among those aged 65 and older, the prevalence of hypertension was 16.17% for that age group, and patients in this age group were 72.6 times more likely to have higher odds of hypertension compared to those aged 20–29 (the reference group). Similarly, patients aged 45–64 and 30–44 had 26.4 times and 6.83 times higher odds of having hypertension, respectively, compared to the reference group, reflecting a clear trend of increasing odds with age.

Female patients had a 4.963% prevalence rate of hypertension among all females in the state and had 1.10 times had slightly higher odds of hypertension than male patients (the reference group). Although White patients represented the largest proportion of the patient population, Black patients had a higher prevalence rate (7.143%) and had 1.51 times higher odds of hypertension compared to White patients. Native American patients also had higher odds (OR = 1.10), whereas Hispanic (OR = 0.38), Asian (OR = 0.27), and ‘Other’ racial groups (OR = 0.05) had significantly lower odds of hypertension compared to White patients.

### Health-related social determinants and prevalence rates

#### Transportation methods and hypertension

The relationship between transportation methods and hypertension prevalence was explored using Pearson correlation and effect estimate analyses (Table 2). Driving to work showed a significant positive correlation with hypertension prevalence (*r* = 0.137, *p* = 0.001) and an effect estimate (EE) of 1.065 (95% CI: 1.027–1.106), indicating a higher effect estimate of hypertension prevalence among individuals who drive to work. Conversely, reliance on taxicabs (*r* = −0.151, *p* < 0.001; EE = 0.772, 95% CI: 0.673–0.885), walking to work (*r* = −0.173, *p* < 0.001; EE = 0.879, 95% CI: 0.829–0.933), and working without a car (*r* = −0.127, *p* = 0.002; EE = 0.913, 95% CI: 0.863–0.967) were significantly associated with a lower prevalence of hypertension. Public transit use and having no vehicle in the household were not significantly correlated with hypertension prevalence.

**Table 2.** Correlation and effect estimates for social determinants of health variables

Variables	Pearson correlation	*p* > |t|	Effect estimate	EE 95% CI lower	EE 95% CI upper
**Percentage of transportation method**					
Drive to work	0.137	0.001*	1.065	1.027	1.106
No vehicle in family	0.040	0.333	1.022	0.978	1.067
Public transit to work	0.029	0.485	1.035	0.940	1.140
Taxicab to work	−0.151	<0.001*	0.772	0.673	0.885
Walk to work	−0.173	<0.001*	0.879	0.829	0.933
Work with no car	−0.127	0.002*	0.913	0.863	0.967
**Percentage of income**					
Income under 10,000	0.089	0.029*	1.081	1.008	1.160
Income under 14,999	0.187	<0.001*	1.233	1.128	1.347
Income under 24,999	0.189	<0.001*	1.148	1.083	1.216
Income under 49,999	0.124	0.002*	1.057	1.020	1.096
Income under 99,999	−0.126	0.002*	0.941	0.905	0.978
Income over 100,000	−0.135	0.001*	0.967	0.948	0.986
**Food resources per 1000 people**					
Convenience store	−0.309	<0.001*	0.638	0.571	0.714
Fast food restaurant	−0.316	<0.001*	0.799	0.757	0.844
Full-service restaurant	−0.331	<0.001*	0.761	0.715	0.810
Supermarket/grocery store	−0.312	<0.001*	0.549	0.474	0.636
**Percentage of insurance type**					
Medicare	0.034	0.403	1.010	0.987	1.034
Medicaid	0.177	<0.001*	1.071	1.039	1.105
Private insurance	−0.187	<0.001*	0.944	0.921	0.967
Self-pay	0.004	0.927	1.006	0.893	1.132
Other insurance	0.021	0.614	1.017	0.953	1.085
**Percentage of food stamp**					
Unmarried received food stamp	0.164	<0.001*	1.033	1.017	1.050
Households received food stamp	0.252	<0.001*	1.112	1.076	1.150
Received food stamp below poverty line	0.227	<0.001*	1.179	1.114	1.248
No food stamp below poverty line	−0.077	0.059	0.949	0.899	1.002
With public assistance income/ food stamp	0.258	<0.001*	1.113	1.078	1.150

Mean values were significantly different from zero (correlation) from the null effect (logistic regression): *p < 0.01.

EE (Effect Estimate per 1% increase); CI, confidence interval.

Superscript symbol (*) denotes significant relationships at the level of *p* < 0.01.

#### Income levels and hypertension

Income-related variables revealed distinct patterns of association with hypertension prevalence. Lower income brackets were positively correlated with prevalence, with income under $10,000 showing a significant positive correlation (*r* = 0.089, *p* = 0.029; EE = 1.081, 95% CI: 1.008–1.160). Similarly, income under $14,999 and $24,999 exhibited stronger associations (*r* = 0.187, *p* < 0.001; EE = 1.233, 95% CI: 1.128–1.347 and *r* = 0.189, *p* < 0.001; EE = 1.148, 95% CI: 1.083–1.216, respectively). Higher income brackets, such as income over $99,999 (*r* = −0.126, *p* = 0.002; EE = 0.941, 95% CI: 0.905–0.978) and income over $100,000 (*r* = −0.135, *p* = 0.001; EE = 0.967, 95% CI: 0.948–0.986), were associated with lower odds of hypertension prevalence, underscoring the protective association of higher income levels.

#### Access to food resources

Neighbourhood access to food resources showed a consistent relationship with hypertension prevalence. Higher density of convenience stores, fast food restaurants, and grocery stores per 1,000 people was negatively correlated with hypertension prevalence, with effect estimate below 1 (e.g., supermarkets: *r* = −0.312, *p* < 0.001; EE = 0.549, 95% CI: 0.474–0.636). This suggests that increased availability of healthy food options, such as supermarkets, may help reduce hypertension prevalence.

#### Insurance coverage and hypertension

Insurance type demonstrated varying relationships with hypertension prevalence. Medicaid coverage was associated with a significant positive correlation (*r* = 0.177, *p* < 0.001; EE = 1.071, 95% CI: 1.039–1.105), while private insurance showed a significant negative correlation (*r* = −0.187, *p* < 0.001; EE = 0.944, 95% CI: 0.921–0.967). Other insurance types, such as Medicare and self-pay, were not significantly associated with hypertension prevalence.

#### Food stamps and hypertension

The relationship between food stamp usage and hypertension prevalence showed a consistent pattern of positive associations for most categories. Unmarried individuals receiving food stamps were significantly more likely to have hypertension (*r* = 0.164, *p* < 0.001; EE = 1.033, 95% CI: 1.017–1.050). Households receiving food stamps also exhibited higher odds (*r* = 0.252, *p* < 0.001; EE = 1.112, 95% CI: 1.076–1.150), as did individuals receiving food stamps below the poverty line (*r* = 0.227, *p* < 0.001; EE = 1.179, 95% CI: 1.114–1.248). In contrast, individuals below the poverty line without food stamps showed a borderline significant negative association (*r* = −0.077, *p* = 0.059; EE = 0.949, 95% CI: 0.899–1.002), suggesting that lack of food assistance in this population may exacerbate health disparities. Finally, individuals with public assistance income or food stamps also demonstrated higher odds of hypertension (*r* = 0.258, *p* < 0.001; EE = 1.113, 95% CI: 1.078–1.150).

**Figure 1.**
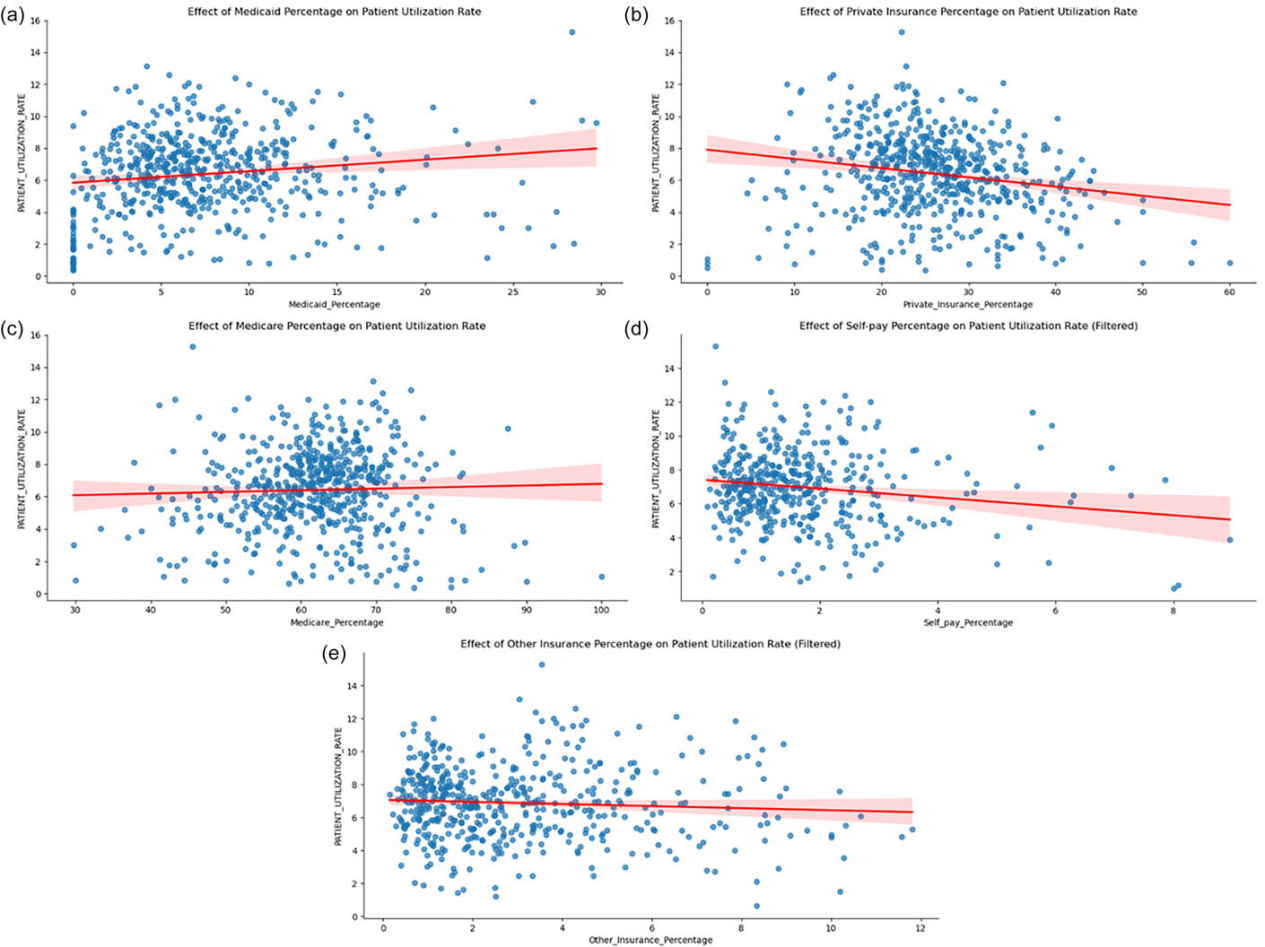
Relationship between insurance coverage types and patient prevalence rate. Scatter plots illustrate the association between the percentage of various insurance coverage types and patient prevalence rates. Each plot represents linear regression analysis with 95% confidence intervals (shaded areas). Panels depict insurance types individually: (a) Medicaid, (b) Private Insurance, (c) Medicare, (d) Self-pay, and (e) Other Insurance. Patient prevalence rates reflect the percentage of patients diagnosed with hypertension.

Some social determinant factors such as transportation time, insurance type, and food stamp relief, etc., exhibited weaker or non-significant correlations, reflecting the multifaceted nature of these relationships and underscoring the need for further investigation to fully elucidate their impact on hypertension prevalence. We utilised various data visualisations to better represent the insurance-related findings at the state level. As shown in Figure 1, each insurance type’s association with the hypertension prevalence rate is illustrated. In Figure 1a, Medicaid coverage shows a significant positive correlation with the prevalence rate, with most zip codes having a Medicaid-insured population ranging from approximately 3% to 10%. Similarly, Medicare coverage in Figure 1c demonstrates a slightly positive correlation with the prevalence rate, with the majority of zip codes having 50% to 75% of patients using Medicare as their insurance type.

Private insurance, as shown in Figure 1b, is the second-largest insurance category among the zip codes, generally ranging from 15% to 40%. It exhibits a significant negative correlation on the prevalence rate, which aligns with our earlier Pearson correlation findings. Figure 1d displays the results for self-pay patients – those without any healthcare insurance, who account for about 1% to 3% of patients at the zip code level. We excluded zip codes with 0% self-pay patients to prevent skewed results. Figure 1e focuses on patients using ‘Other Healthcare Insurance’. Similar to the self-pay category, we excluded zip codes with 0% AND outliers that could potentially bias the results. This ‘other insurance’ group generally constitutes 1% to 4% of total insurance holders in a zip code and shows a slightly negative correlation on the prevalence rate.

### Milwaukee county prevalence rate

While the statewide analysis provided valuable insights into the relationships between social determinants of health and hypertension prevalence, examining these patterns at a more localised level offers an opportunity to uncover unique regional trends and disparities. Milwaukee County, as one of the most populous and diverse areas in the state, presents an ideal setting to explore how neighbourhood-level factors, including access to resources, transportation, income, and healthcare, influence hypertension prevalence within a concentrated geographic region. The following section explores Milwaukee County-specific data, highlighting key differences from the broader statewide trends and providing a deeper understanding of localised health disparities.

### Patients’ distribution based on income and race

**Figure 2.**
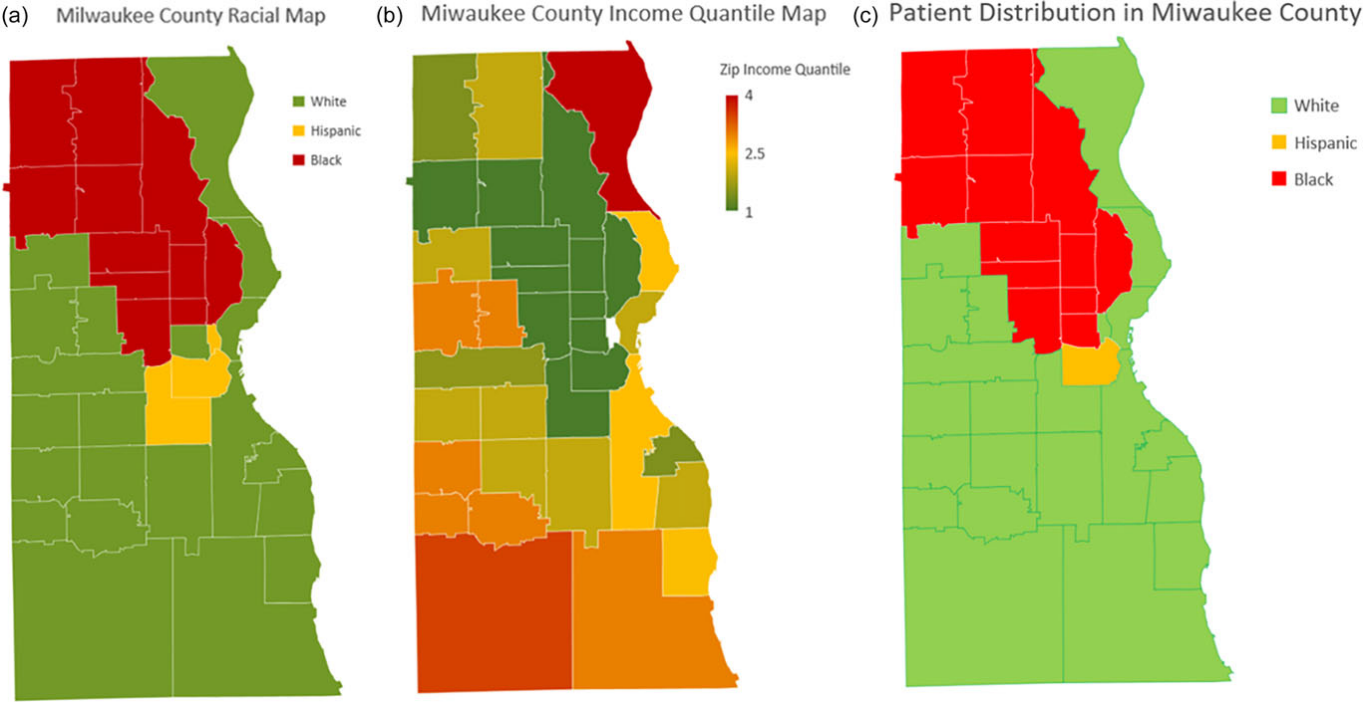
Demographic and socioeconomic characteristics across Milwaukee County by ZIP code region. Maps illustrate variations in racial composition, socioeconomic status, and patient demographic distribution within Milwaukee County. Panel (a) shows racial composition, indicating majority populations categorised as White, Hispanic, or Black across ZIP codes. Panel (b) displays the ZIP-code-level median household income quantiles, ranging from low (green, quantile 1) to high (red, quantile 4). Panel (c) illustrates patient demographic distribution by racial majority, categorised similarly to panel (a).

Milwaukee County demonstrates a diverse distribution of racial and economic groups; Figure 2 illustrates the racial distribution in Milwaukee County. The northwest region of the county is predominantly Black, while the central area has a significant Hispanic population. The remaining areas are predominantly White, covering much of the southern and eastern parts of the county.

The income distribution across Milwaukee County is shown in Figure 2b. Higher-income areas are concentrated in the southern and eastern neighbourhoods, overlapping with predominantly White regions. Conversely, the northwest and central areas, home to larger Black and Hispanic populations, are predominantly in the lowest income quartiles.

**Figure 3.**
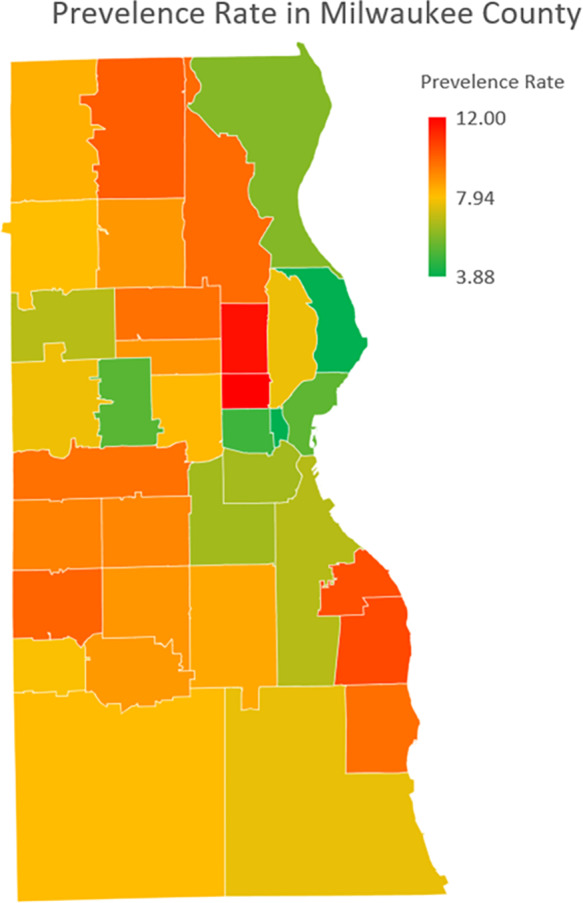
Hypertension prevalence rate (%) across Milwaukee County by ZIP code region. The choropleth map depicts the geographic distribution of hypertension prevalence rates across ZIP code regions within Milwaukee County. Prevalence rates are colour-coded, ranging from low (green) to high (red), with specific numerical breakpoints indicated in the colour legend. Hypertension prevalence rates represent the percentage of patients diagnosed with hypertension within each ZIP code region.

This demographic and socioeconomic segregation is linked to hypertension prevalence rates shown in Figure 3. Areas with the highest prevalence rates (shaded in red) overlap with the northwest neighbourhoods, predominantly Black, and the central areas, predominantly Hispanic. These areas, marked by lower income levels, report prevalence rates of hypertension as high as 12%. In contrast, neighbourhoods in the southern and eastern regions, associated with higher income levels and predominantly White populations, have significantly lower prevalence rates, often below 4%. The spatial visualisation underscores the disparities in hypertension prevalence, demonstrating its strong association with both racial distribution and income levels across Milwaukee County (Figure 2a and c).

### Food resource distribution in Milwaukee County

#### Convenience stores

**Figure 4.**
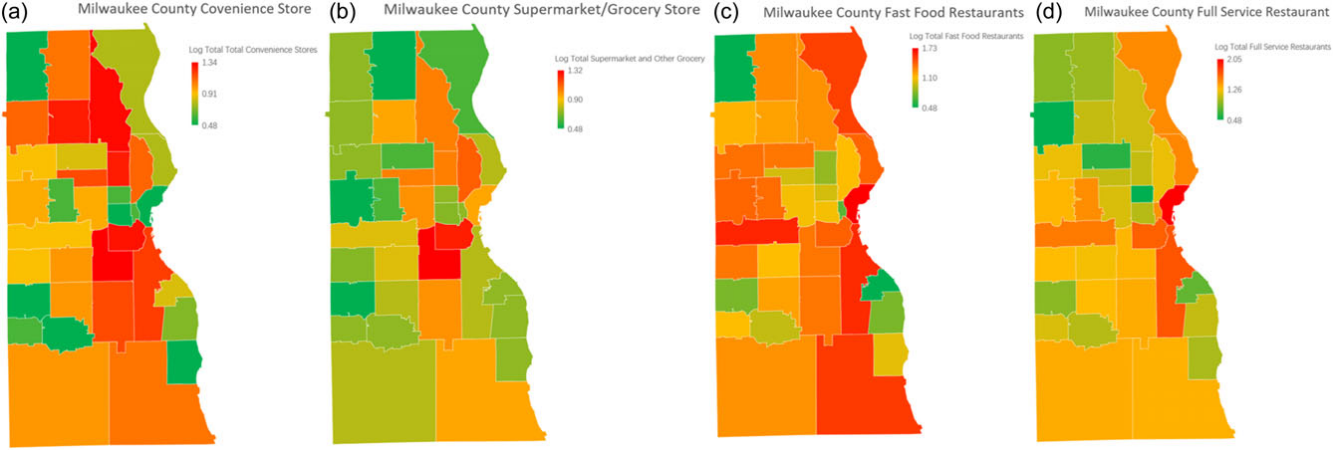
Geographic distribution of food resources across Milwaukee County by ZIP code region. Maps depicting the distribution of various food resources normalised per 1000 people and log transformed. Panel (a) illustrates the distribution of convenience stores, panel (b) shows supermarkets and grocery stores, panel (c) represents fast food restaurants, and panel (d) illustrates full-service restaurants. Colour gradients represent resource density from low (green) to high (red).

The distribution of convenience stores per 1000 people (Figure 4a) reveals a dense concentration in the northwest and central regions of Milwaukee County, areas predominantly occupied by Black and Hispanic populations and characterised by lower income levels. These regions also report the highest prevalence rates of hypertension. The clustering of convenience stores in these neighbourhoods reflects the limited access to healthier food options, potentially contributing to the heightened rates of chronic diseases like hypertension.

#### Supermarkets and grocery stores (in the centre of the county, redo writing)

Supermarkets and grocery stores per 1000 people (Figure 4b) are more sparsely distributed in the northwest and central areas, which are home to Black and Hispanic populations and correspond to lower-income neighbourhoods. In contrast, suburban neighbourhoods in the southern and eastern parts of the county, predominantly occupied by White populations with higher income levels, have greater access to supermarkets and grocery stores. These disparities align with lower hypertension prevalence rates in higher-income areas, often below 4%, further emphasising the role of socioeconomic and racial factors in shaping health outcomes.

#### Fast food

The distribution of fast-food restaurants in Milwaukee County follows the pattern of major highways and urban centres, as illustrated in Figure 4c. Areas with a higher density of fast-food establishments are concentrated along key transportation routes and within central urban neighbourhoods. These regions often correspond to neighbourhoods with higher hypertension prevalence rates, which exceed 12% in certain areas. Conversely, areas with fewer fast-food establishments, typically located in suburban and rural parts of the county, exhibit significantly lower hypertension prevalence rates, often below 4%. This spatial alignment suggests a potential link between the availability of fast-food options and hypertension prevalence. This emphasise the need to consider the food environment when addressing public health concerns in Milwaukee County.

#### Full-service restaurants

The availability of full-service restaurants per 1000 people (Figure 4(d)) is notably higher in the southern and eastern suburbs of Milwaukee County, which are predominantly White and higher-income areas. Conversely, the central and northwest neighbourhoods, marked by lower income levels and higher proportions of Black and Hispanic populations, have fewer full-service dining options. The lack of full-service restaurants and the reliance on convenience and fast food in these areas likely contribute to the elevated hypertension prevalence rates observed in these regions (Figure 3).

#### Summary of food resources and hypertension prevalence

The analysis of food resources across Milwaukee County and the broader state highlights a critical link between food accessibility and hypertension prevalence. While the statistical results from Table 2. provide a statewide perspective, the heatmaps of Milwaukee County reveal localised patterns that reinforce these findings. Areas with higher food accessibility, whether convenience stores, supermarkets, grocery stores, full-service restaurants, or fast-food establishments consistently show lower hypertension prevalence rates. Conversely, neighbourhoods with limited food options exhibit significantly higher rates of hypertension. These patterns underscore the detrimental impact of food deserts, where residents face limited access to healthy food resources. However, even outside of food deserts, individuals with low incomes may still experience limited food accessibility due to economic barriers, further compounding their risk for hypertension. This challenge of living in a food desert or having insufficient financial resources to access available food options, creates significant public health disparities.

## Discussion

In our population-based epidemiological investigation of social determinants of health and hypertension prevalence across Wisconsin, we identified several statistically significant associations with important implications for both clinical practice and public health. Utilising an integrated socioecological analytical framework, our analysis revealed complex and multidimensional relationships among environmental exposures, socioeconomic conditions, and hypertension outcomes. These findings advance current knowledge by demonstrating patterns of association that extend beyond the linear relationships typically described in prior studies, underscoring the importance of accounting for broader contextual factors in understanding hypertension risk.

### Demographic comparison

To assess the representativeness of our findings, we compared Wisconsin-based prevalence estimates with global and U.S. benchmarks reported in recent analyses. Globally, hypertension affects 1 in 3 adults, and the number of people living with hypertension doubled between 1990 and 2019, reaching approximately 1.3 billion in 2019. In the United States, data from the 2021–2023 National Health and Nutrition Examination Survey (NHANES) indicate that 47.7% of adults had hypertension, with prevalence higher in men (50.8%) than women (44.6%).^(17,18)^ Within Wisconsin, our inpatient sample demonstrated an overall prevalence of 6.0%, a lower estimate that corresponds to the clinical setting rather than population-based surveillance. Nonetheless, the demographic gradients observed locally are consistent with national patterns. Female patients in Wisconsin exhibited a prevalence of 4.96%, markedly lower than the global estimate for women. At the same time, adults aged 65 years and older accounted for more than half of all cases, with a prevalence of 16.2%. These comparisons highlight both the methodological differences between datasets and the importance of situating state-level findings within a broader global context.

### Cultural and ethnic dietary patterns

Particularly noteworthy was the identification of a distinct pattern wherein Hispanic patients in central Milwaukee County exhibited significantly lower hypertension rates despite socioeconomic disadvantages comparable to other low-income populations. This finding extends the established ‘Hispanic paradox’ in cardiovascular mortality documented by previous researchers,^(1,3)^ who demonstrated lower cardiovascular mortality rates among Hispanics despite higher prevalence of cardiovascular risk factors and lower socioeconomic status.

The protective pattern observed in our study population may be related to traditional Mexican dietary practices, which are characterised by higher consumption of specific foods previously reported to be associated with favourable cardiovascular outcomes. Recent phytochemical analyses of traditional Mexican foods have identified substantial concentrations of bioactive compounds with reported associations to cardiovascular benefits.^(19)^ A comprehensive analysis by Alatorre-Cruz et al.^(20)^ documented significant concentrations of phenolic compounds in traditional Mexican diets, confirming their potent antioxidant and anti-inflammatory properties. These compounds have been associated with modulating multiple pathophysiological pathways implicated in hypertension development, including endothelial dysfunction, oxidative stress, and vascular inflammation.^(13,16)^

Traditional Mexican diets, which include staple foods such as beans, corn, chili peppers, and a variety of indigenous plant products, have been widely associated with beneficial cardiovascular profiles and lower blood pressure.^(21,22)^ These dietary associations extend beyond individual food items, as preparation methods such as nixtamalization, fermentation, and other traditional cooking practices may contribute to the availability and use of nutrient-rich components.^(23–25)^

More broadly, the predominance of plant-based proteins, dietary fibre, and complex carbohydrates within these diets, alongside relatively lower consumption of ultra-processed foods, aligns with nutritional patterns that have been consistently linked to lower hypertension prevalence in observational and intervention studies.^(26)^ This dietary composition aligns with nutritional factors previously associated with lower hypertension prevalence, including adequate potassium intake, moderate sodium consumption, and abundant antioxidant compounds.

In addition to the Hispanic paradox, our analysis also revealed relatively low hypertension prevalence among Asian and Native American patients in Milwaukee County. For Asian populations, this observation is consistent with recent evidence showing that adherence to dietary patterns emphasising fruits, vegetables, and whole grains has been associated with lower risk of incident hypertension, with South Asian Americans experiencing a 67% reduction when closely following a DASH-style diet.^(27)^ Such a protective association may partly reflect broader dietary traditions rich in plant-based proteins, legumes, and soy, which have been consistently associated with lower blood pressure in both observational studies and dietary intervention trials.^(28)^ At the same time, scholars caution against treating Asian American populations as a single group; dietary practices are highly diverse and shaped by cultural, social, and structural factors,^(29)^ suggesting that the relatively low prevalence observed locally should be interpreted with attention to subgroup variation.

Native American patients also showed comparatively lower prevalence in our cohort, a result that appears consistent with emerging evidence on culturally tailored dietary practices. Recent community-based interventions that provided tribally administered healthy food boxes reported associations with lower blood pressure and improved diet quality among Native American adults.^(30,31)^ A protocol currently underway, the Native Opportunities to Stop Hypertension (NOSH) trial, similarly integrates a culturally adapted DASH diet to support cardiovascular health among American Indian and Alaska Native adults.^(32)^ These findings support protective associations between culturally responsive dietary strategies and hypertension prevalence, even in the face of broader socioeconomic disadvantages.

### Income

Income level is an important factor associated with hypertension prevalence, with higher-income groups consistently exhibiting lower rates. In neighbourhoods with median and high incomes, access to a greater quantity and variety of food resources is associated with lower hypertension prevalence.^(33–35)^ These communities’ benefit from increased purchasing power, which not only allows residents to prioritise healthier food choices and adopt nutritious dietary behaviours, ^(35,36)^ but also is associated with greater access to medical resources and treatments.^(37)^ This advantage is associated with early diagnosis and timely medical intervention, which is further associated with improved cardiovascular health outcomes.

In contrast, low-income neighbourhoods face compounding challenges that are associated with a higher prevalence of hypertension. Economic constraints can limit individuals’ ability to access healthy food options, even in areas with adequate food resources. Although food deserts are traditionally associated with rural settings, they are also prevalent in urban environments, disproportionately affecting low-income communities. The patterns observed in West Milwaukee County align with existing research,^(22)^ which reports that urban food deserts often overlap with neighbourhoods characterised by elevated rates of chronic disease and health inequities.^(6)^ While these areas may have enough food outlets, lower-than-average household incomes hinder residents’ ability to access fresh and nutritious food options. This economic barrier is often associated with reliance on less healthy, calorie-dense, and processed foods, which are associated with higher hypertension prevalence.^(36)^

### Healthcare access beyond insurance coverage

In our insurance analysis, we revealed only modest associations. Medicaid coverage showed a positive and statistically significant association (EE = 1.071, 95% CI: 1.039–1.105, *p* < 0.001), while private insurance demonstrated a negative and significant association (EE = 0.944, 95% CI: 0.921–0.967, *p* < 0.001). Other forms of coverage, including Medicare, self-pay, and ‘other’, did not reach significance. This uneven pattern may indicate that insurance type alone does not fully capture the barriers patients face, and it may reflect that additional dimensions of healthcare access must be considered.

One critical dimension is geographic accessibility. Prior studies have demonstrated that patients living closer to primary care providers have been observed to show higher awareness of their hypertension and higher rates of control, while those in underserved or segregated areas have been observed with lower rates of awareness and control.^(38,39)^ These findings imply that insurance coverage may be insufficient when the spatial distribution of healthcare providers restricts the ability of patients to engage with care.

However, Geographic access cannot be separated from system capacity and appointment availability. Even where providers are physically present, long delays before specialty or follow-up appointments remain common, with some patients waiting several months.^(40)^ Evidence also suggests that shorter waiting times are associated with greater engagement and adherence, indicating that timeliness is associated with more effective chronic disease management.^(41)^ Thus, the benefits of insurance are contingent not only on the presence of providers but also on the system’s ability to accommodate patients in a timely manner.

A further consideration is continuity of care. Hypertension outcomes have been associated with patients maintaining regular follow-up visits.^(42)^ Experiences during the COVID-19 pandemic showed that continuity maintained through telemedicine was associated with better blood pressure control.^(43)^ These observations indicate that the stability of patient-provider relationships has been associated with more consistent hypertension management, while fragmented or episodic care may be associated with less effective management of hypertension despite insurance coverage.

### Food resource and access

Access to sufficient and diverse food resources is an important factor associated with hypertension prevalence. This study demonstrates a consistent negative association between food resource availability and hypertension prevalence across Milwaukee County and Wisconsin as a whole. Regardless of the type of food resources, whether convenience stores, supermarkets, fast-food outlets, or full-service restaurants, greater accessibility was associated with lower prevalence rates of hypertension (Table 2). These findings underscore the association between food accessibility and chronic disease patterns, particularly hypertension, through its association with dietary diversity and healthier eating practices.^(44,45)^

Food deserts, defined as areas with a limited number of food resources regardless of type, were predominantly identified in neighbourhoods with high hypertension prevalence rates.^(5,6,46)^ These areas often lack sufficient food outlets, such as supermarkets or grocery stores, which corresponds with the limited residents’ ability to access fresh and nutritious food options.^(5,23,26)^ Conversely, regions with a concentration of highly processed and calorie-dense food sources, such as convenience stores and fast-food outlets, may be associated with poor dietary habits and increased hypertension prevalence.^(24,26)^

The heatmaps (Figure 4) visually depict these contrasting patterns across Milwaukee County. In the northwest region, despite having an adequate number of convenience stores and fast-food outlets, the lack of supermarkets and grocery stores corresponds with limited access to fresh fruits and vegetables and is associated with higher hypertension prevalence rates.^(21,22)^ This imbalance in food resource types highlights the association of food quality and diversity in shaping dietary health outcomes. On the other hand, the southern parts of Milwaukee County, which have fewer food resources overall, also exhibit high hypertension prevalence rates. This emphasises the importance of total food resource density in relation to health outcomes, irrespective of food type.^(45,47,48)^

### Food assistance paradox

Contrary to anticipated associations, individuals receiving food stamps consistently exhibited higher hypertension prevalence compared to demographically similar individuals without such assistance (EE = 1.179, 95% CI: 1.114–1.248 for those below the poverty line with food stamps versus EE = 0.949, 95% CI: 0.899–1.002 for those below the poverty line without assistance). Although these programmes are designed to alleviate food insecurity, our findings suggest they may not adequately address nutritional quality. This gap may lead to increased reliance on energy-dense, nutrient-poor foods, potentially contributing to the higher observed prevalence of hypertension among participants. This observation aligns with research by Zhang et al.^(49)^ documenting associations between food stamp participation and suboptimal dietary patterns among low-income women.

Among low-income populations, food stamp recipients represent a distinct subgroup facing unique challenges in accessing and utilising nutritious food options. Economic constraints further compound these challenges, as affordability often takes precedence over nutritional value. This economic trade-off has associated with continued reliance on less nutritious foods, which in turn has been associated with elevated hypertension prevalence rates.^(24,50)^

These dietary patterns among food stamp recipients have been reported in relation to adverse health outcomes, particularly hypertension. The over-reliance on processed, calorie-dense foods and the insufficient intake of fruits, vegetables, and nutrient-rich alternatives have been associated with increased chronic disease risk.^(12)^ Poor nutritional intake is associated with higher prevalence of hypertension risk factors over time, highlighting potential areas for improving nutritional considerations within food assistance programmes. Policy reforms prioritising access to affordable, nutrient-dense foods, coupled with public health initiatives promoting balanced diets, may have relevance for reducing the health disparities experienced by food stamp recipients.^(49)^

### Urban change and gentrification

Beyond programmatic barriers, neighbour-level changes may be associated with changes in access in complex ways. Urban redevelopment may be associated with greater physical proximity to food outlets and other amenities, yet at the same time, it may be associated with lower affordability and accessibility for long-standing residents. Evidence indicates that such improvements are not consistently associated with healthier diets. A natural experiment in a public housing community showed that proximity to a newly opened supermarket was not associated with better dietary outcomes among children, except for those who lacked reliable transportation, highlighting that mobility is associated with differences in access.^(51)^ Similarly, recent analyses caution that some neighbourhoods contain supermarkets or other outlets that are physically nearby but remain economically inaccessible. These areas can be understood as a variant of food deserts, sometimes described in the literature as ‘food mirages’, where healthier choices remain out of reach for many households.^(52)^ Our findings are consistent with this pattern, indicating that purchasing power may be associated with whether environmental interventions align with reduced hypertension prevalence.

When viewed through the lens of gentrification, these dynamics become even more pronounced. Redevelopment may be associated with changes in the retail mix and neighbourhood amenities, while also being associated with higher costs and reduced access for long-standing residents. Systematic reviews describe how gentrification has been associated with variants of food deserts, with healthier retailers introduced but at price points that exclude lower-income households.^(53)^ Large-scale analyses across U.S. cities have reported associations between gentrification, which may also coincide with worsening social deprivation and reduced life expectancy among Black and Hispanic residents, despite some improvements in health care availability.^(54)^ Similar findings have been noted with green gentrification, where new parks and walkable spaces are used more often by newcomers than by long-term residents, indicating associations with continued patterns of exclusion.^(55)^ These observations indicate that associations between urban change and health outcomes may relate less to the simple presence of new infrastructure and more to whether those resources remain affordable and accessible to the communities they are meant to serve.

### Behavioural confounders

Behavioural factors also form a crucial part of the broader risk landscape, and their association with hypertension is equally important as geographic, socioeconomic, and environmental determinants. Hypertension is associated not only with the neighbourhoods where individuals live but also with the routines and practices embedded in daily life. Evidence from randomised clinical trials shows that lifestyle modifications, including weight reduction, regular exercise, and reduced alcohol intake, can significantly lower blood pressure.^(56)^ These findings indicate the importance of considering behavioural risk factors and structural exposures as interdependent rather than separate domains.

Physical activity provides one of the clearest examples of this connection. Population-based analyses have shown that higher-intensity activity is associated with reduced risk of hypertension, even after accounting for overall energy expenditure.^(57)^ Yet activity patterns are not evenly distributed; they are often associated with work demands, neighbourhood safety, and access to recreational spaces, indicating that community context may be associated with individual behaviour. Stress adds another dimension. Large-scale studies using both survey data and biological markers demonstrate that individuals experiencing sustained occupational stress have a higher prevalence of hypertension.^(58)^ Stress exposures have also been observed more often in communities facing economic instability, which are associated with heightened risks created by poverty and residential disadvantages.

Tobacco use further illustrates how individual practices intersect with social patterning. Experimental studies report associations with short-term blood pressure elevations after smoking, and longitudinal evidence confirms elevated long-term risk among chronic smokers.^(59)^ Finally, alcohol consumption has been associated with a largely linear relationship with blood pressure, with no clear safe threshold.^(60)^ Because drinking patterns vary across income and racial groups in the United States, alcohol use may be associated with reinforcing or widening the disparities observed in our Wisconsin population, due to the state’s long-standing brewing industry and cultural emphasis on alcohol availability.

### From food deserts to food environment synergy

Recent studies have worked on characterising neighbourhood food environments through binary classifications of ‘food deserts’ or ‘food secure’ areas.^(5,6)^ The Food Environment Policy Index^(61)^ and the exposome concept^(62)^ have provided valuable frameworks for examining how physical food access is associated with health outcomes. However, these approaches have significant limitations. They often examine environmental factors in isolation, focusing primarily on the physical presence or absence of food resources without adequately addressing the economic and cultural dimensions that are associated with food choices. They do not capture the complex, synergistic interactions between multiple environmental factors that collectively are associated with cardiovascular health outcomes.

In this project, we propose a novel conceptual framework – the ‘Food Environment Synergy Model’ that better captures these complex interactions. Unlike traditional models, our framework integrates three critical dimensions that are often studied separately: food resource distribution (physical access), economic accessibility (affordability), and cultural dietary patterns (nutritional knowledge and practices). By combining these factors, our approach reveals how their interactions shape unique patterns of risk and resilience within communities.

For instance, neighbourhoods with high physical access to food resources but limited economic accessibility demonstrate different risk patterns than those with both physical and economic barriers. Similarly, areas with strong cultural dietary traditions, such as those maintaining traditional Mexican dietary practices, exhibit unique resilience patterns that may be associated with variations in the expected socioeconomic health gradient. These patterns illustrate how social determinants rarely operate independently but combine in ways that are associated with layered profiles of risk and resilience. Recent scholarship supports this view, showing that affordability, availability, and cultural norms intersect to be associated with dietary behaviours in low-income contexts,^(63)^ that structural inequities combine with cultural practices to be associated with food choices,^(64)^ and that nutrition disparities are best understood through frameworks capturing multi-level interrelationships.^(65)^ Together with work by Kebede et al.^(66)^ which emphasises food systems as complex socioecological systems, these perspectives are consistent with the Food Environment Synergy Model’s premise that physical, economic, and cultural determinants must be considered jointly to capture the complexity of community health environments.

The Food Environment Synergy Model helps frame the seemingly paradoxical findings in our study, such as the protective associations observed in Hispanic populations despite economic disadvantages, and the higher hypertension rates among food stamp recipients compared to similarly impoverished non-recipients. By moving beyond unidimensional classifications of food environments, this model provides a more nuanced understanding of how environmental factors collectively are associated with cardiovascular health outcomes and points toward more targeted intervention approaches.^(61)^ Our model supports recent calls for integrated assessment approaches that consider multiple interacting factors in food environment research.^(67)^

In operational terms, the Food Environment Synergy Model may indicate the relevance of interventions that address multiple dimensions of access simultaneously. At the policy level, this may include subsidies for fresh produce in underserved neighbourhoods, incentives for culturally relevant food retailers, and adjustments to programmes such as SNAP to better support healthy purchasing. At the community level, strategies could combine nutritional education with financial support like vouchers and partnerships with cultural organisations that reinforce protective dietary traditions. Together, these approaches provide examples of how the model can be translated into practice, and they highlight a pathway for public health planning that moves beyond single-dimensional solutions toward more integrated and context-sensitive strategies.^(68)^

### Limitations

Several methodological limitations warrant acknowledgment. First, our cross-sectional design precludes definitive causal interpretations regarding the temporal relationships between social determinants and hypertension development. Second, while our spatial mapping methodology provides valuable insights into geographic distributions of risk factors, it potentially masks individual-level heterogeneity within ZIP code boundaries. Third, our utilisation of hospitalisation data introduces potential selection bias by capturing only individuals who accessed hospital services, potentially underrepresenting those with undiagnosed hypertension or limited healthcare access. Fourth, while we identified cultural dietary patterns as potentially protective among Hispanic populations, we lack detailed nutritional intake data to quantitatively characterise the specific dietary components that may be related to the observed cardioprotective associations. Finally, it should be noted that food environments are dynamic and can change rapidly over time, whereas our data captures a single time point, limiting the extent to which findings capture longitudinal changes in food access and availability.

### Strengths and future research directions

Despite these limitations, our study offers several methodological strengths. The substantial sample size (387,047 patients) and extensive geographic coverage (597 ZIP codes) provide robust statistical power and ecological validity. Additionally, our approach to distinguishing between food deserts and food swamps offers a more nuanced characterisation of food environment association with hypertension prevalence than conventionally employed literature. The integrated socioecological analytical framework enabled simultaneous examination of multiple environmental and socioeconomic determinants at both individual and community levels, highlighting interaction patterns that may not be evident in narrower analyses.

Future research should pursue several promising trajectories based on our findings. Longitudinal studies tracking changes in neighbourhood food environments, transportation patterns, and hypertension outcomes could strengthen causal inferences and identify critical intervention windows. Detailed nutritional analyses employing metabolomic and phytochemical methodologies could isolate specific bioactive compounds in traditional Mexican diets that confer cardiovascular protection, potentially identifying novel nutraceutical compounds for targeted hypertension prevention. Implementation research could evaluate the feasibility and effectiveness of incorporating cultural dietary knowledge into clinical and community-based hypertension prevention programmes. Additionally, economic analyses of food assistance programme modifications prioritising nutritional quality could quantify potential healthcare cost savings from reduced hypertension prevalence.

## Conclusion

Hypertension prevalence in Wisconsin is associated with a complex interplay of socioeconomic, environmental, and cultural factors. Our findings indicate that low-income populations, particularly those living in food deserts or relying on food stamps, face heightened risks that are associated with limited access to diverse and nutritious food options, which may be associated with unhealthy dietary patterns. In contrast, higher-income communities show patterns associated with improved food access, healthier diets, and superior medical resources, which correspond to lower observed prevalence rates. Notably, cultural dietary practices, as observed among Hispanic patients, may be associated with lower hypertension prevalence despite economic disadvantages, underscoring the potential protective associations of traditional eating habits. These findings highlight the relevance of considering food resource availability, the nutritional quality of food assistance programmes, and cultural as well as socioeconomic contexts in public health planning. However, because this study is cross-sectional, causal interpretations cannot be made, and longitudinal studies are needed to clarify temporal relationships and underlying mechanisms.
